# Diagnosing Ocular Surface Squamous Neoplasia in East Africa: Case-Control Study of Clinical and In Vivo Confocal Microscopy Assessment

**DOI:** 10.1016/j.ophtha.2013.09.027

**Published:** 2014-02

**Authors:** Marie B. Nguena, Jan G. van den Tweel, William Makupa, Victor H. Hu, Helen A. Weiss, Stephen Gichuhi, Matthew J. Burton

**Affiliations:** 1 Department of Ophthalmology, Kilimanjaro Christian Medical Centre, Moshi, Tanzania; 2 Department of Pathology, University Medical Centre, Utrecht, The Netherlands; 3 International Centre for Eye Health, Faculty of Infectious and Tropical Diseases, London School of Hygiene & Tropical Medicine, London, United Kingdom; 4 Department of Infectious Disease Epidemiology, Faculty of Epidemiology and Population Health, London School of Hygiene and Tropical Medicine, London, United Kingdom; 5 Department of Ophthalmology, University of Nairobi, Nairobi, Kenya

## Abstract

**Objective:**

To examine the reliability of clinical examination and in vivo confocal microscopy (IVCM) in distinguishing ocular surface squamous neoplasia (OSSN) from benign conjunctival lesions.

**Design:**

Case-control study.

**Participants:**

Sixty individuals with conjunctival lesions (OSSN and benign) and 60 age-matched controls with normal conjunctiva presenting to Kilimanjaro Christian Medical Centre, Moshi, Tanzania.

**Methods:**

Participants were examined and photographed, and IVCM was performed. Patients with conjunctival lesions were offered excisional biopsy with histopathology and a human immunodeficiency virus (HIV) test. The IVCM images were read masked to the clinical appearance and pathology results. Images were graded for several specific features and given an overall categorization (normal, benign, or malignant). A group of 8 ophthalmologists were shown photographs of conjunctival lesions and asked to independently classify as OSSN or benign.

**Main Outcome Measures:**

Comparison of the histopathology diagnosis with the clinical and IVCM diagnosis.

**Results:**

Fifty-two cases underwent excisional biopsy with histopathology; 34 were on the OSSN spectrum, 17 were benign, and 1 was lymphoma. The cases and controls had comparable demographic profiles. Human immunodeficiency syndrome infection was more common in OSSN compared with benign cases (58.8% vs. 5.6%; odds ratio, 24.3, 95% confidence interval [CI], 2.8–204; *P =* 0.003). Clinically, OSSN lesions more frequently exhibited feeder vessels and tended to have more leukoplakia and a gelatinous appearance. Overall, the ophthalmologists showed moderate agreement with the histology result (average kappa = 0.51; 95% CI, 0.36–0.64). The masked grading of IVCM images reliably distinguished normal conjunctiva. However, IVCM was unable to reliably distinguish between benign lesions and OSSN because of an overlap in their appearance (kappa = 0.44; 95% CI, 0.32–0.57). No single feature was significantly more frequent in OSSN compared with benign lesions. The sensitivity and specificity of IVCM for distinguishing OSSN from benign conjunctival lesions were 38.5% and 66.7%, respectively.

**Conclusions:**

In East Africa, conjunctival pathology is relatively common and can present significant diagnostic challenges for the clinician. In this study, neither clinical examination nor IVCM was found to reliably distinguish OSSN from benign conjunctival pathology because of an overlap in the features of these groups. Therefore, IVCM cannot currently replace histopathology, and management decisions should continue to rely on careful clinical assessment supported by histopathology as indicated.

Ocular surface squamous neoplasia (OSSN) is the most common malignant ocular surface disease in Africa.^1^, ^2^, ^3^ It ranges from small areas of conjunctiva intra-epithelial neoplasia (CIN) to large invasive squamous cell carcinoma (SCC).^4^ There has been a marked increase in the incidence of this disease in East Africa over the last couple of decades, attributed to human immunodeficiency virus (HIV)/AIDS.^3^, ^5^, ^6^, ^7^ The disease now presents at a younger age than previously and affects more women than men. Although HIV positivity is a well-defined major risk factor for OSSN, approximately half of all cases in East Africa are HIV positive and the role of other etiologic factors needs to be defined.^4^, ^7^ Data from case-control studies suggest that damage from ultraviolet light exposure in equatorial regions may be important.^7^ Human papilloma virus has been postulated to be involved, but its contribution remains uncertain despite several studies.^8^

One of the challenges facing clinicians managing OSSN is distinguishing early neoplastic from benign lesions (Fig 1A, B). This is a particular problem in East Africa, where benign conjunctival changes (pterygium, pingeculum) are relatively common. There is probably an overlap in the clinical features of these different benign lesions and OSSN, with no signs being exclusive to OSSN, although this has not been formally evaluated. Some features have been reported to be more common in advanced cases of OSSN.^4^ Histopathology is necessary to confirm a diagnosis of OSSN. However, pathology services are generally scarce in East Africa outside larger centers. As a result, many lesions are excised without pathologic analysis.

**Figure 1:**
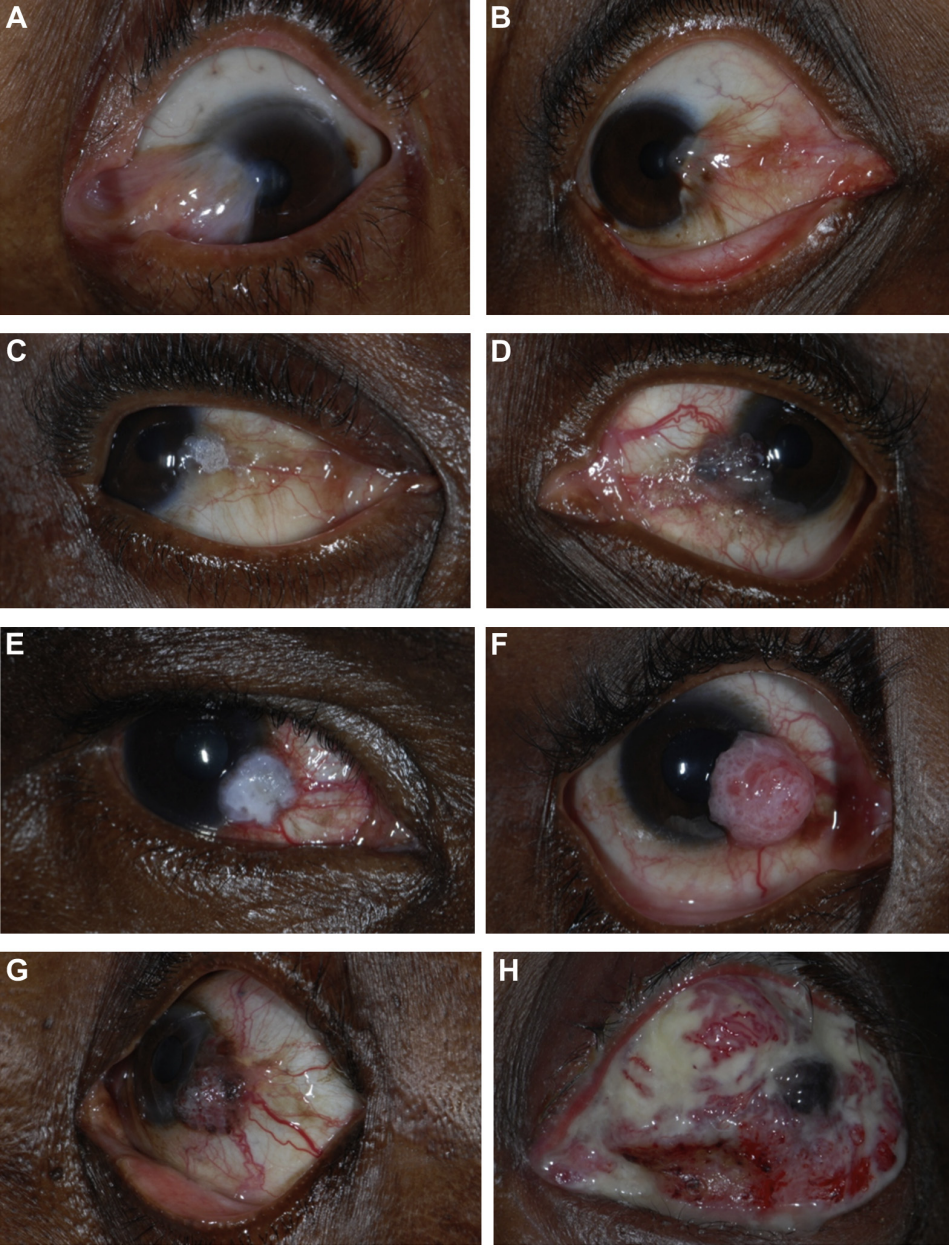
Photographs of a pterygium and various grades of ocular surface squamous neoplasia: pterygium **(A)**, conjunctiva intra-epithelial neoplasia (CIN) 1 **(B)**, CIN 3 **(C)**, carcinoma in situ **(D)**, squamous cell carcinoma (SCC) grade 1 **(E)**, SCC grade 2 **(F)**, SCC grade 3 **(G)**, and SCC with orbital invasion **(H)**.

The standard approach for treating suspected OSSN is an excisional biopsy, often with cryotherapy to the conjunctival margin and sometimes with topical chemotherapy.^9^, ^10^ An initial incisional biopsy is usually performed for larger lesions to make a diagnosis to plan treatment. In recent years, there has been a move to consider primary topical chemotherapy for suspected OSSN without a tissue diagnosis.^11^ Given the potential uncertainty of diagnosis based on clinical signs alone, several groups have investigated whether in vivo confocal microscopy (IVCM) can help distinguish cases of OSSN.^12^, ^13^, ^14^, ^15^, ^16^ These reports of relatively few cases (26 in the whole literature) have tended to conclude that IVCM may be helpful in making a diagnosis of OSSN. Several specific features, such as irregular cell size, hyper-reflectivity, prominent bright nucleoli (“starry night appearance”), and mitotic figures, have been singled out as potential markers for OSSN.^13^

We report a prospective study of patients with conjunctival lesions presenting to an ophthalmic unit in Tanzania. We investigate the diagnostic accuracy of the clinical examination and whether certain features help to distinguish OSSN from benign lesions. Second, we evaluate the utility of IVCM in identifying OSSN in a masked study of conjunctival lesions.

## Methods

### Ethical Approval

This study adhered to the tenets of the Declaration of Helsinki. It was reviewed and approved by the Kilimanjaro Christian Medical Centre (KCMC) Ethics Committee, Tanzania.

### Study Design and Participants

In this case-control study, we compared individuals with conjunctival lesions (“cases,” either benign or OSSN) with individuals with clinically normal conjunctiva (“controls”). Recruitment of both cases and controls was conducted at the Department of Ophthalmology, KCMC Hospital, Moshi, Tanzania, from May to November 2011. Cases were consecutive individuals presenting with a conjunctival lesion that on clinical grounds required surgical excision. For each case, we recruited a control presenting for a condition that was not affecting the ocular surface and who had healthy conjunctiva on slit-lamp examination (e.g., cataract). These control participants were age-matched to the corresponding case (±5 years). All participants were aged 18 years or older. The study was explained to eligible study subjects, and written informed consent was obtained before enrollment. All enrolled patients with conjunctival lesions received counseling and were offered testing for HIV, which is standard of care at KCMC. Patients with a positive HIV test result were referred to the Comprehensive Treatment Center.

### Clinical Assessment

Patients with conjunctival lesions were asked about symptoms (ocular pain, irritation, itchiness, redness, decreased vision, proptosis, and discharge). Participants were asked where they lived within Tanzania and their main occupation. The surface of the eye was carefully examined with a slit lamp for conjunctival pathology. We recorded lesion location and size and the presence of specific features, including fibrovascular tissue (Fig 1A, B), leukoplakia (Fig 1C), gelatinous appearance (Fig 1D), feeder vessels (Fig 1D), papilliform structure (Fig 1F), limbal involvement, and orbital invasion (Fig 1H). The lesions were photographed using a digital single lens reflex camera with a macro lens.

### In Vivo Confocal Microscopy Examination

In vivo confocal microscopy examination of the bulbar conjunctiva was performed using the Heidelberg Retina Tomograph 3 in combination with the Rostock Corneal Module (Heidelberg Engineering GmbH, Dossenheim, Germany). A sterile, disposable polymethylmethacrylate cap was mounted on the head of the confocal microscope, and a drop of topical anesthetic (proparacaine 0.5%) was instilled into the inferior fornix of the eye before the examination. For cases, the lesion and surrounding areas were scanned. For controls, the temporal or nasal inter-palpebral conjunctiva was scanned. Representative images of the examined conjunctiva were obtained using the “volume scan” mode, which collects a series of 40 parallel images 2 μm apart; both surface (starting at the epithelium) and deeper sets of scans were performed.

**Figure 2:**
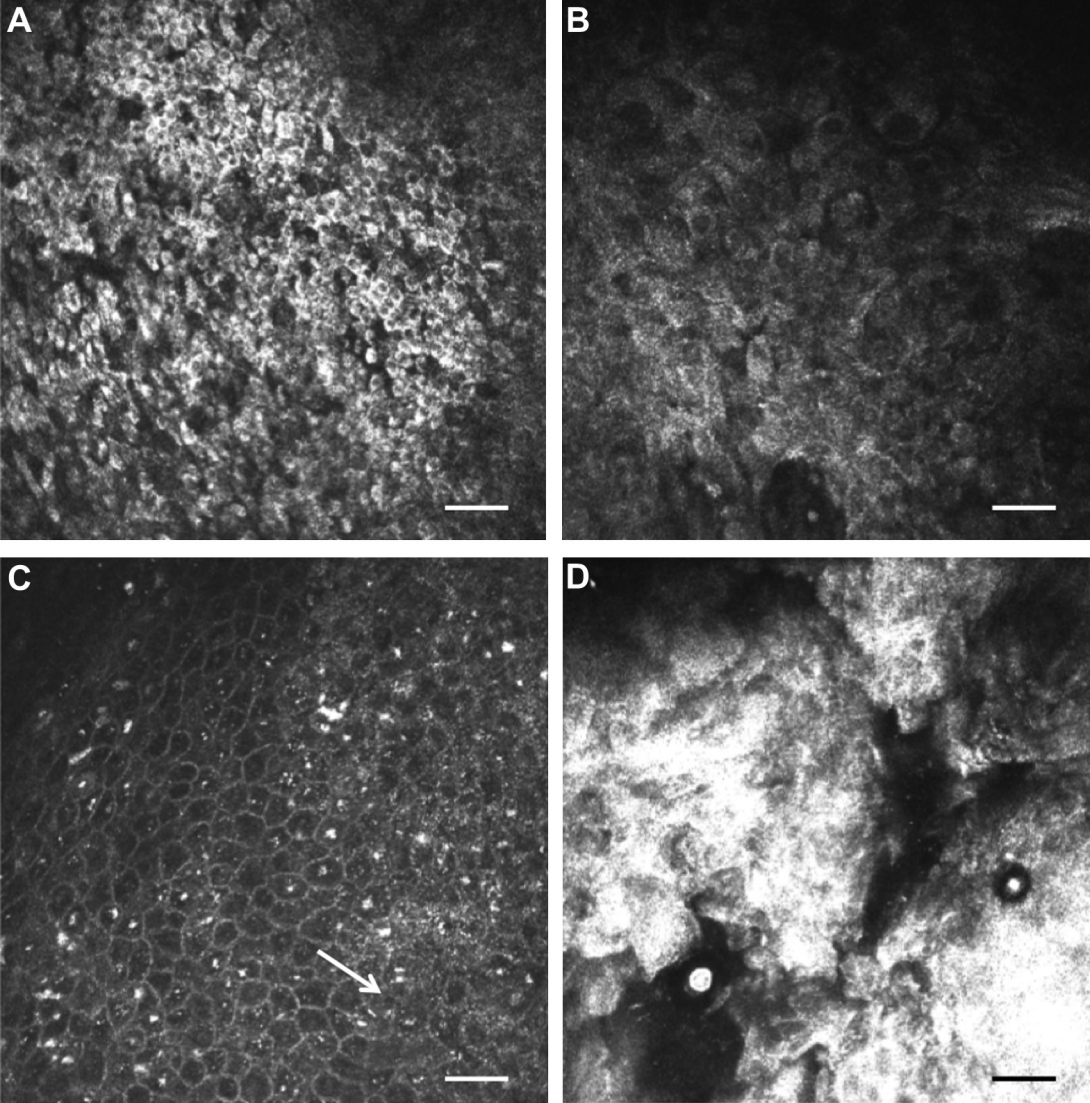
In vivo confocal microscopy images of conjunctival ocular surface squamous neoplasia. **A,** Small hyper-reflective cells. **B,** Large hyper-reflective cells of variable size. **C,** Possible mitotic figure (*arrow*) and prominent basal cell nucleoli. **D,** Amorphous, possibly keratinized material. Scale bar 50 μm.

All confocal images were graded over a 2-day period, after recruitment had been completed. Two ophthalmologists (M.B.N., M.J.B.) jointly examined all the images; they were masked to the case/control status, clinical features, and histopathology results. The grading of the images was based on separately examining each of the 40 parallel images in each of the volume scan stacks. The following features, which are said to be potentially discriminating in previous studies, were evaluated: variable epithelial cell size, hyperreflectivity, presence of mitotic cells, and prominent bright nucleoli (“starry night appearance”) (Fig 2).^13^ An overall provisional categorization based on the IVCM findings was made: (1) normal, (2) abnormal benign, or (3) abnormal malignant.

### Surgical Treatment

Lesions were treated surgically. Small to medium-sized lesions that were clinically suspicious for OSSN were fully excised using the “no touch technique” with a 3- to 4-mm margin of healthy-looking tissue. Surgical sponges saturated with 5-fluorouracil were applied to the excision site for 5 minutes followed by irrigation with normal saline. 5-Fluorouracil treatment was not used if the case was thought to be benign pathology, such as a pterygium. Incisional biopsies were performed for large lesions to obtain tissue for histology assessment. If malignant pathology was confirmed, radical surgery (enucleation or exenteration) was then offered to the patient. Biopsy tissue was not collected from the normal controls.

### Histopathology

Biopsy specimens from the cases were immediately placed in 10% formaldehyde solution. These were mounted in paraffin wax, and 5-μm sections were cut perpendicular to the conjunctival surface and stained with hematoxylin–eosin. All specimens were examined by a single pathologist (J.G.T.). Ocular surface squamous neoplasia was classified using the standard definitions for mild (CIN I), moderate (CIN II), and severe (CIN III) dysplasia, carcinoma in situ, and invasive SCC.^17^ Squamous cell carcinoma was subdivided by the degree of differentiation: well (I), moderate (II), and poor (III).

### Clinical Diagnosis Study

To evaluate the ability of clinicians to correctly distinguish between benign and malignant conjunctival lesions, we showed a group of 8 ophthalmologists (3 consultants and 5 residents) a series of 43 photographs of lesions for which a histologic diagnosis also was available by the time this component was conducted. This included a mixture of benign and neoplastic pathologies. They were asked to independently write down their diagnosis and whether they thought it was benign or malignant.

### Data Analysis

Data were entered into Access 2007 (Microsoft Corp., Redmond, WA) and analyzed using Stata 11.0 (StataCorp LP, College Station, TX). Demographic characteristics of cases and controls were compared by conditional logistic regression. For the purpose of analysis, occupation was condensed to “indoors” or “outdoors” and region of residence to “Kilimanjaro region” and “outside Kilimanjaro region.” The Wilcoxon rank-sum test was used to compare the ages between cases and control because the distribution was skewed. The frequency of clinical signs and symptoms in benign and neoplastic lesions was compared using the Fisher exact test.

The Kappa statistic was used to assess the agreement between the categorized tissue diagnosis (benign or OSSN), and clinical diagnosis made from photographs by the 8 ophthalmologists (benign or OSSN). To calculate an average value, the kappa statistics for each grader were transformed to *Z* scores using the Fisher *Z* transformation, averaged, and then back-transformed to a kappa statistic. The clinical and IVCM features were analyzed in relation to the histologic diagnosis. Associations between the tissue diagnosis and the presence of specific clinical or IVCM features were assessed by the Fisher exact test. Agreement between the categorized tissue diagnosis (normal, benign, or malignant) and the masked IVCM grading (normal, benign, or malignant) was assessed by a weighted Kappa statistic.

## Results

### Study Participants

Sixty individuals with conjunctiva lesions (cases) and 60 age-matched controls with healthy ocular surfaces were recruited. The cases and controls had comparable demographic profiles: age, sex, region of origin, and occupational history (Table 1).

**Table 1:** Demographic Characteristics and Diagnoses of Study Participants

Characteristics	Cases	Controls	Total	*P* Value∗
	N = 60	N = 60		
Female	30 (50%)	36 (60%)	66 (55%)	0.28
Region (Kilimanjaro)	35 (58.3%)	39 (65.0%)	74 (61.6%)	0.45
Occupation (outdoor)	35 (58.3%)	30 (50.0%)	65 (54.1%)	0.34
Age (yrs)				
Mean	43.5	43.7	43.6	0.95
Range	23–80	21–75	21–80	
Histologic diagnosis				
CIN I	2 (3.3%)	–	–	
CIN II	3 (5.0%)	–	–	
CIN III	5 (8.3%)	–	–	
CIS	5 (8.3%)	–	–	
SCC I	12 (20.0%)	–	–	
SCC II	4 (6.6%)	–	–	
SCC III	3 (5.0%)	–	–	
Lymphoma	1 (1.6%)	–	–	
Pterygium	5 (8.3%)	–	–	
Pingueculum	3 (5.0%)	–	–	
Papilloma	3 (5.0%)	–	–	
Other benign lesions	6 (10.0%)	–	–	
No histopathology	8 (13.3%)	–	–	
Control diagnosis				
Normal examination	–	26 (43.3%)	–	
Diabetic retinopathy	–	10 (16.6%)	–	
Cataract	–	9 (15.0%)	–	
Glaucoma	–	3 (5.0%)	–	
Astigmatism	–	6 (10.0%)	–	
Hyperopia	–	1 (1.6%)	–	
Presbyopia	–	3 (5.0%)	–	
Optic atrophy	–	1 (1.6%)	–	
Myopia	–	1 (1.6%)	–	

CIN = conjunctiva intra-epithelial neoplasia; CIS = carcinoma in situ; SCC = squamous cell carcinoma.

∗ *P* value from conditional logistic regression for sex, region, and occupation and from Wilcoxon rank-sum test for age.

### Diagnoses

Five cases declined surgical excision of their lesions, and a further 3 cases had surgical excision without histopathology, leaving 52 of 60 cases with a histologic diagnosis (Table 1). For 34 cases, the histologic diagnosis was on the OSSN spectrum, 17 had benign lesions, and 1 case had lymphoma. Of the 8 cases without histology, the clinical diagnosis was OSSN in 6, pterygium in 1, and pingeculum in 1. Biopsy tissue was not collected from control participants with clinically normal conjunctiva. The most common clinical diagnoses of controls were normal examination results, diabetic retinopathy, and cataract (Table 1).

### Symptoms and Signs

Among individuals with conjunctival lesions, there was no difference in the frequency of various symptoms between benign and OSSN cases (Table 2). However, OSSN cases had a shorter time to presentation, suggesting a more symptomatic or rapidly evolving lesion (3.7 vs. 8.8 months; *P =* 0.03). Several clinical signs were more frequently found in eyes with OSSN (with borderline statistical significance): feeder vessel, leukoplakia, and gelatinous appearance (Fig 1, Table 2). A fibrovascular appearance was more frequent among benign lesions. The OSSN lesions tended to be larger than benign lesions, although this difference was not significant (Wilcoxon rank-sum *P =* 0.8).

**Table 2:** Symptoms and Clinical Signs by Histologic Diagnosis

	OSSN Cases	Benign Cases∗	*P* Value†
Symptom			
Irritated red eye	21 (61.7%)	8 (44.4%)	0.26
Discharge	20 (58.8%)	11 (61.1%)	1.00
Foreign body sensation	14 (41.1%)	10 (55.5%)	0.39
Decreased vision	8 (23.5%)	3 (16.6%)	0.73
Pain	5 (14.7%)	5 (27.7%)	0.29
Proptosis	5 (14.7%)	1 (5.5%)	0.65
Itchy	2 (5.8%)	1 (5.5%)	1.00
Mean duration (mos, 95% CI)	3.7 (2.4–5.8)	8.8 (3.9–19)	0.029
Sign			
Feeder vessel	30 (88.2%)	11 (61.1%)	0.034
Limbal involvement	26 (76.4%)	9 (50.0%)	0.07
Leukoplakia	15 (44.1%)	3 (16.6%)	0.07
Gelatinous	13 (38.2%)	2 (11.1%)	0.055
Pedunculated	4 (11.7%)	2 (11.1%)	1.00
Papilliform	2 (5.8%)	1 (5.5%)	1.00
Orbital invasion	2 (5.8%)	0 (0.0%)	0.54
Fibrovascular tissue	1 (2.9%)	7 (44.4%)	0.001

CI = confidence interval; OSSN = ocular surface squamous neoplasia.

∗ Including 1 lymphoma case.

† *P* values calculated using Fisher exact test for variables except duration, which was calculated by Wilcoxon rank-sum test.

### Human Immunodeficiency Virus and Conjunctival Lesions

Among the 52 cases (benign and OSSN) with a tissue diagnosis, there was a strong association between HIV and OSSN (58.8%) compared with benign cases (5.6%) (odds ratio, 24.3; 95% confidence interval [CI], 2.8–204; *P =* 0.003) (Table 3). Human immunodeficiency virus infection also tended to be more frequent in people with SCC compared with CIN (68.4% vs. 46.7%; *P =* 0.2).

**Table 3:** Human Immunodeficiency Virus Test Results by Tissue Diagnosis of Conjunctival Lesions

Tissue Diagnosis	HIV Positive	OR	95% CI	*P* Value
Benign lesions∗	1/18 (5.6%)	1	–	–
CIN	7/15 (46.7%)	14.9	1.56–142	0.019
SCC	13/19 (68.4%)	36.8	3.93–344	0.002

CI = confidence interval; CIN = conjunctiva intra-epithelial neoplasia; HIV = human immunodeficiency virus; OR = odds ratio; SCC = squamous cell carcinoma.

∗ Including 1 lymphoma case.

### Reliability of the Clinical Diagnosis

Eight ophthalmologists were shown a consecutive series of 43 images of conjunctival lesions and asked to classify them into 2 broad categories of benign and neoplastic. Their diagnoses were compared with the histopathology results, and kappa statistics were calculated: average kappa statistic 0.51 (95% CI, 0.36–0.64). Two ophthalmologists had “fair” agreement (kappas of 0.21 and 0.40), 3 ophthalmologists had “moderate” agreement (0.41–0.60), and 3 ophthalmologists had “substantial” agreement (0.61–0.80). The correct diagnosis was made in 56% to 86% of the cases.

### In Vivo Confocal Microscopy

In vivo confocal microscopy was attempted on all 120 cases and controls, and the scans were graded in a masked manner. Three controls were excluded because of inadequate IVCM images. Histopathology results were available for 52 of 60 cases. The archived IVCM images from 8 of 52 of the cases were not of sufficient quality for masked grading. Therefore, the analysis of IVCM was limited to 101 participants: 44 cases (with both histopathology and adequate scans) and 57 controls.

The IVCM images were examined for the presence of specific features: hyper-reflective cells, variation of cell size, mitotic cells, and “starry night” appearance of the basal layer (Fig 2, Table 4). Several lesions showed marked surface changes on IVCM with extensive, hyper-reflective material with large irregular cellular structure. A possible mitotic cell was observed in only 1 lesion (Fig 2C). Among the 57 controls with IVCM images, 3 had some hyper-reflective cells and 2 showed some variation in epithelial cell size. For each of the graded features, there were statistically significant differences in their frequency between the normal controls and cases (benign and malignant combined). However, we found no evidence for a difference in the frequency of these IVCM features between benign and malignant conjunctival lesions.

**Table 4:** In Vivo Confocal Microscopy Features by Disease Group, Based on Masked Grading

IVCM Features	Controls	Benign Cases∗	OSSN Cases	*P* Value†	*P* Value‡
	N/57 (%)	N/18 (%)	N/26 (%)		
Hyper-reflective cells				<0.001	0.9
None	54 (94.7%)	7 (38.3%)	12 (46.1%)		
<50%	3 (5.3%)	7 (38.8%)	8 (30.7%)		
>50%	0 (0.0%)	4 (22.2%)	6 (23.1%)		
Variation in cell size				<0.001	0.5
Uniform	55 (96.4%)	9 (50%)	17 (65.3%)		
Mild	1 (1.7%)	6 (33.3%)	4 (15.3%)		
Marked	1 (1.7%)	3 (16.7%)	5 (19.2%)		
Starry night appearance of basal cells				0.001	0.8
Nil	57 (100%)	14 (77.7%)	22 (84.6%)		
Few to many	0 (0.0%)	2 (11.1%)	1 (3.8%)		
Amorphous material	0 (0.0%)	2 (11.1%)	3 (11.5%)		

IVCM = in vivo confocal microscopy; OSSN = ocular surface squamous neoplasia.

∗ Including 1 lymphoma case.

† *P* value is for the comparison of normal controls with all cases (benign and malignant combined) by Fisher exact test.

‡ *P* value is for the comparison of benign with malignant cases, by Fisher exact test.

Each set of IVCM images was given an overall classification by the masked readers: normal, benign abnormal, or malignant. The comparison of this overall IVCM classification with the histopathology diagnosis is shown in Table 5. Agreement was found to be moderate with an unweighted kappa score of 0.44 (95% CI, 0.32–0.57). The linear weighted kappa score was 0.54 (95% CI, 0.40–0.67). The sensitivity and specificity of IVCM for distinguishing OSSN from benign conjunctival lesions were 38.5% (95% CI, 0.21–0.59) and 66.7% (95% CI, 0.41–0.86), respectively.

**Table 5:** Comparison of the Overall Classification from the In Vivo Confocal Microscopy Based on Masked Grading (Normal, Benign, or Malignant) versus the Final Histologic Diagnosis

Tissue Diagnosis	IVCM Diagnosis			Total
	Normal	Abnormal Benign	Malignant	
Normal control	50	7	0	57
Benign lesion	4	8	6	18
Malignant lesion	5	11	10	26
Total	59	26	16	101

IVCM = in vivo confocal microscopy.

Grey shading highlights where there was agreement.

## Discussion

Timely detection and treatment of OSSN are challenges in East Africa, with many patients only presenting when the lesion is already established. The reasons for this are many: Access to eye care services outside the major urban centers is generally limited; therefore, patients may delay traveling for treatment until the lesion is more obvious and symptomatic. When patients do present, there can be further delay in making the diagnosis of OSSN because initially it may be thought to be a benign lesion. Delayed or missed diagnosis can have considerable effects on ocular morbidity and even mortality.

In this study based in an East African ophthalmic referral center, OSSN was a common cause of conjunctival pathology, with presentation rates comparable to those in previous reports from this unit.^3^, ^4^ Time between onset of symptoms and presentation was shorter for OSSN than for benign lesions, possibly because OSSN develops more rapidly. However, some individuals with OSSN had noticed a problem many months earlier. Strategies are needed to raise general awareness that growths on the eye need prompt attention. This is particularly important in people who have a known diagnosis of HIV, but this needs to be done in a way that minimizes stigma.

There is an overlap in the clinical signs of (early) OSSN and some benign lesions (Fig 1A, B). However, a number of features tended to be more frequent in cases of OSSN: feeder vessels, gelatinous appearance, and leukoplakia. These and other signs could form the basis of a diagnostic algorithm to assist the clinician in identifying OSSN cases, and they warrant further study in a larger data set. Overall, the 8 ophthalmologists, most of whom had seen many cases of OSSN previously, achieved only moderate agreement with the histologic diagnosis. There was a wide range in their diagnostic accuracy, suggesting that if the key features that point to a diagnosis of OSSN can be identified and emphasized in training, there should be scope to improve the detection rate.

The prevalence of HIV infection in OSSN cases was comparable to that in previous studies from the region.^4^ Human immunodeficiency virus seems to be a risk factor for developing more severe disease, being more frequent among cases with SCC than CIN. There was no difference in the time between the onset of symptoms and the presentation between SCC and CIN, suggesting that HIV infection permits a more rapid progression in the disease.

In vivo confocal microscopy is a potentially useful tool in the management of several external eye diseases. It has been particularly helpful in the diagnosis of acanthamoeba and filamentary fungal keratitis.^18^ We have also found the technique informative in the study of conjunctival scarring in trachoma.^19^ In the present study, masked analysis of IVCM clearly distinguished between normal conjunctiva and lesions (benign or malignant). A few clinically normal controls had some minor changes on IVCM, which might be attributable to solar radiation damage. In contrast, it was not possible to reliably distinguish between OSSN and benign conjunctival lesions because there seems to be an overlap in the IVCM features of these various conditions. We did not identify any features that were exclusive to OSSN. Of particular note was the finding that there were several cases of OSSN that were classified as normal on IVCM.

Our conclusion that IVCM does not reliably distinguish OSSN from other conjunctival pathology contrasts with earlier reports that suggest it may be possible to use it for this purpose.^12^, ^13^, ^14^, ^15^, ^16^ However, these earlier reports, mostly from Europe (where the disease spectrum is different), have tended to be of relatively few cases. Moreover, with the exception of 1 series of 4 cases, benign lesion controls have not been recruited for comparison. Finally, the assessment of the images was not masked in all but 1 of the previous studies.^16^ Without systematic masked comparison with benign lesions, it is difficult to know how useful the observed features are. Studies of the IVCM appearance of pterygium have reported hyper-reflective epithelial cells, a feature previously said to be associated with OSSN.^20^, ^21^ This is the first time IVCM has been used in Africa to study OSSN. Overall, the disease tends to be more advanced and aggressive in this context, and clinical/IVCM features may show significant variation from studies of Caucasian patients with disease largely unassociated with HIV. Further validation is needed using masked grading of OSSN and other conjunctival pathology in other settings.

### Study Limitations

First, histopathology was not available for all conjunctival lesions because the patient declined surgery or the surgeon did not send a sample for analysis. Second, it was not always possible to obtain IVCM scans of sufficient quality for the masked grading, usually because the patient was unable to tolerate the examination. Third, the masked analysis was limited to the set of stored volume scans; in practice, the formation of a diagnostic impression with IVCM is based on a continuous, dynamic examination in which the examiner is not masked to the clinical appearance of the eye. A limitation of current IVCM technology is that it is not possible to identify exactly where on the lesion the scan is collected from; therefore, it is not possible to put it in exact correspondence with findings from histopathology. Finally, the conclusions of this study may not be generalizable to the use of IVCM to detect OSSN in other settings if the distribution of disease stage is different.

In conclusion, ocular surface squamous neoplasia remains a major clinical challenge in the East African context. A simple and cheap diagnostic test to help distinguish benign from malignant pathology would be of considerable utility in the East African context, where pathology services may not be readily available.
